# Right-sided patent ductus arteriosus with bovine aortic arch, tetralogy of Fallot, dextrocardia and situs inversus

**DOI:** 10.1093/jscr/rjab307

**Published:** 2021-07-23

**Authors:** Khairil Amir Sayuti, Ahmad Mahir Shamsuddin, Ahmad Zuhdi Mamat, Mohd Rizal Mohd Zain, Noraida Ramli, Ariffin Marzuki Mokhtar, Saedah Ali

**Affiliations:** Department of Radiology, School of Medical Sciences, Health Campus, Universiti Sains Malaysia Kubang Kerian, Kelantan, Malaysia; Hospital Universiti Sains Malaysia, Health Campus, Universiti Sains Malaysia, Kubang Kerian, Kelantan, Malaysia; Department of Cardiothoracic Surgery, National Heart Institute, Kuala Lumpur, Malaysia; Department of Surgery, School of Medical Sciences, Health Campus, Universiti Sains Malaysia Kubang Kerian, Kelantan, Malaysia; Hospital Universiti Sains Malaysia, Health Campus, Universiti Sains Malaysia, Kubang Kerian, Kelantan, Malaysia; Department of Pediatrics, School of Medical Sciences, Health Campus, Universiti Sains Malaysia Kubang Kerian, Kelantan, Malaysia; Hospital Universiti Sains Malaysia, Health Campus, Universiti Sains Malaysia, Kubang Kerian, Kelantan, Malaysia; Department of Pediatrics, School of Medical Sciences, Health Campus, Universiti Sains Malaysia Kubang Kerian, Kelantan, Malaysia; Hospital Universiti Sains Malaysia, Health Campus, Universiti Sains Malaysia, Kubang Kerian, Kelantan, Malaysia; Department of Anesthesia, School of Medical Sciences, Health Campus, Universiti Sains Malaysia Kubang Kerian, Kelantan, Malaysia; Hospital Universiti Sains Malaysia, Health Campus, Universiti Sains Malaysia, Kubang Kerian, Kelantan, Malaysia; Department of Anesthesia, School of Medical Sciences, Health Campus, Universiti Sains Malaysia Kubang Kerian, Kelantan, Malaysia; Hospital Universiti Sains Malaysia, Health Campus, Universiti Sains Malaysia, Kubang Kerian, Kelantan, Malaysia

## Abstract

A neonate with cyanosis at birth was found to have a rare type of tetralogy of Fallot. Echocardiography showed dextrocardia, left aortic arch with constricting and tortuous patent ductus arteriosus (PDA). Computed tomography angiography thorax revealed visceroatrial situs inversus, mirror image dextrocardia, tetralogy anatomy and tortuous right PDA arising from bovine brachiocephalic artery. In view of severe cyanosis, emergency division of PDA and modified right Blalock-Taussig shunt through median sternotomy were performed under cardiopulmonary bypass. Post-operatively, the sternum was left open and he developed pulmonary overcirculation requiring prolonged ICU stay. He was discharged well at Day 26 post surgery. This case highlights a rare association tetralogy of Fallot, dextrocardia and situs inversus, with concomitant unilateral right PDA and bovine aortic arch.

## INTRODUCTION

Even though tetralogy of Fallot (TOF) is a prevalent congenital anomaly, there are only few reports of its association with dextrocardia and situs inversus. Having a right-sided patent ductus arteriosus (PDA) has made the situation much complicated for future surgical intervention. Here, we present a case of successful surgical palliation done in this complex anatomy.

## CASE REPORT

A baby boy, born full term with birth weight 3.59 kg via emergency lower segment caesarean section for failed induction of labor at a General Hospital at a neighboring state. He was born with good Apgar score (9^1^10^5^) but later was noted to have mild respiratory distress. Initial chest radiograph showed features consistent with visceral situs inversus and apparent dextrocardia. There was no focal lung lesion. Echocardiography revealed TOF with dextrocardia and a large PDA. He was then discharged home at Day 5 of life with oxygen saturation of 95%. He was readmitted again 3 days later for neonatal jaundice. During the admission, he was noted to have hypercyanotic spell while crying and resolved with knee chest position maneuver. He was started on oral propranolol 0.2 mhg/kg/dose TDS and saturation was maintained ~75–78%. However, the cyanosis was worsening down to 60% and he was intubated and started on prostaglandin infusion. Repeated echocardiography revealed constricting PDA. Due to logistic issue, the child was referred for surgical intervention only at Day 17 of life.

**Figure 1:**
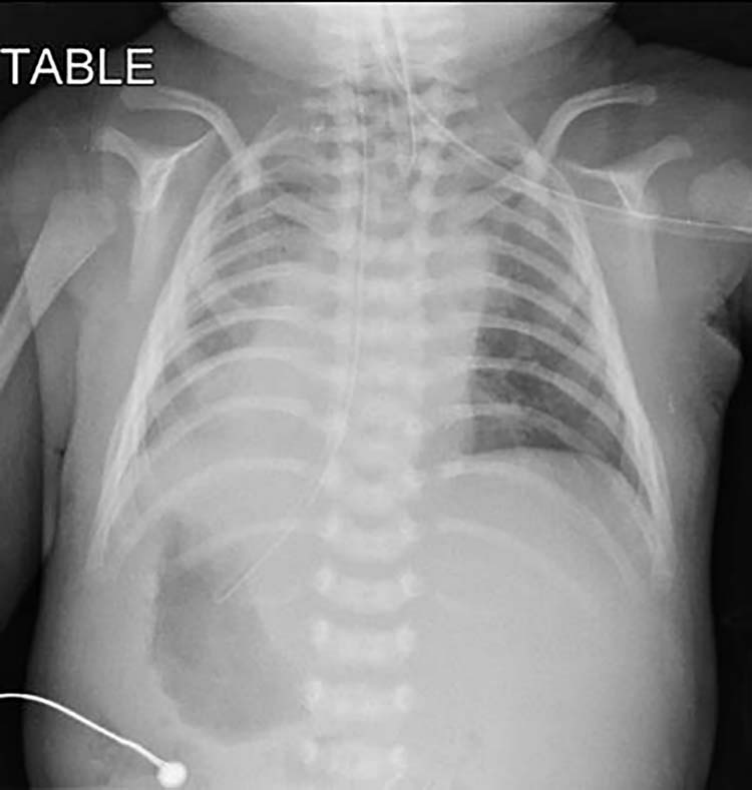
Pre-operative chest radiograph showed apparent dextrocardia and visceral situs inversus (evidenced by lower tip of feeding tube in the right-sided stomach with left-sided homogenous liver opacity).

Upon arrival at our center, he was severely cyanosed, with oxygen saturations of 50–60% on maximum dose of propranolol and prostaglandin infusion. On physical examination, there was an ejection systolic murmur of a grade 3/6. As previously noted the chest radiograph (Fig. 1) revealed apparent dextrocardia with visceral situs inversus. The echocardiography showed tetralogy anatomy, dextrocardia, left aortic arch and severe infundibular stenosis with pressure gradient of 85 mmHg. The PDA appeared constricted, but its origin was not visualized. The pulmonary valve annulus was 4.7 mm (−4 SD) and main pulmonary artery (MPA) was 5.8 mm whilst the size of the branches were within the acceptable limit (−1.0 SD).

**Figure 2:**
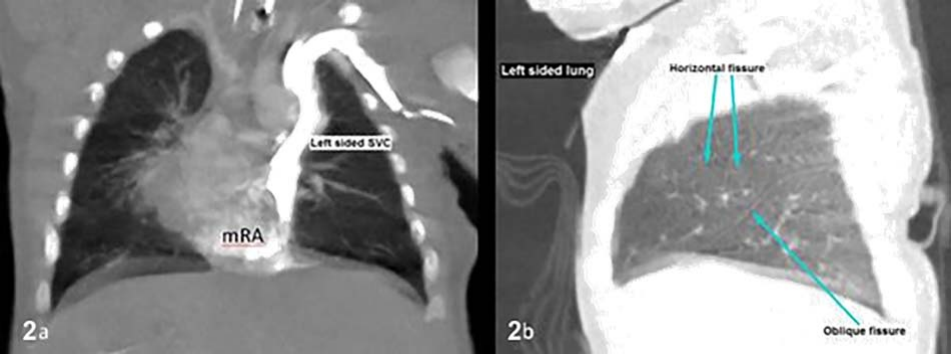
CT thorax confirmed atrial situs inversus whereby there was single left-sided SVC draining into the left-sided mRA as shown on coronal plane (**a**) and trilobed left lung as shown on sagittal plane in lung window (**b**). The pulmonary veins drain into the right-sided mLA (image not shown). Inferior vena cava was poorly demonstrable. SVC, superior vena cava; mRA, morphologic right atrium; mLA, morphologic left atrium.

**Figure 3:**
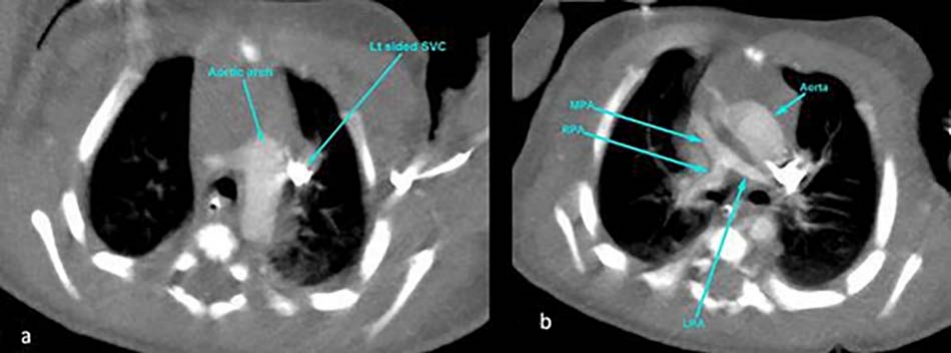
CT angiography thorax showing the left-sided aortic arch (**a**) and inverse relationship between ascending aorta and pulmonary artery. The MPA origin is atretic with hypoplastic RPA and LPA compared with the aorta (**b**). SVC, superior vena cava; RPA, right pulmonary artery; LPA, left pulmonary artery.

**Figure 4:**
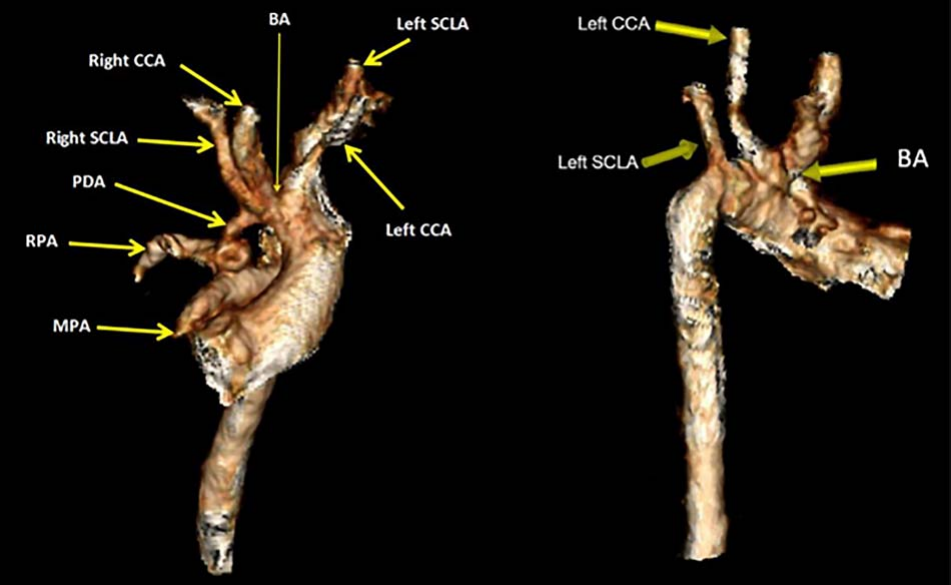
Volume rendered CT images demonstrates bovine aortic arch branches whereby the BA shares a common origin with the left CCA. The left SCLA arises separately as the last branch. The right PDA arises from the BA at pre-bifurcation and it connects inferiorly with the proximal RPA. The inferior end of PDA is tortuous. The MPA showed atretic origin. *BA* brachiocephalic artery, CCA, common carotid artery; SCLA, subclavian artery; RPA, right pulmonary artery.

**Figure 5:**
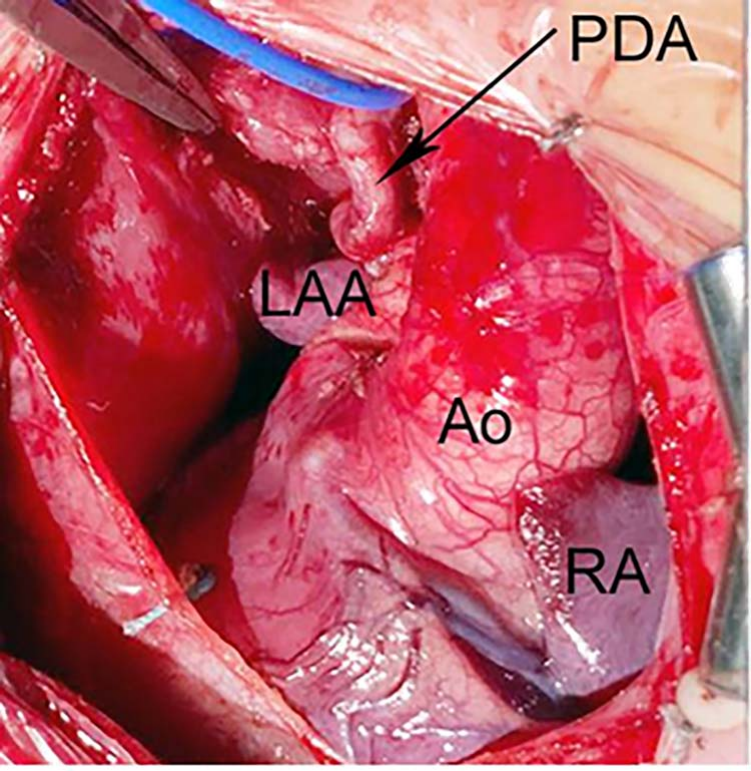
Intraoperative photograph shows dextrocardia with left aortic arch morphology. The PDA is tortous and arising from BA artery. Ao, Aorta; LAA, left atrial appendage; RA, right atrium.

**Figure 6:**
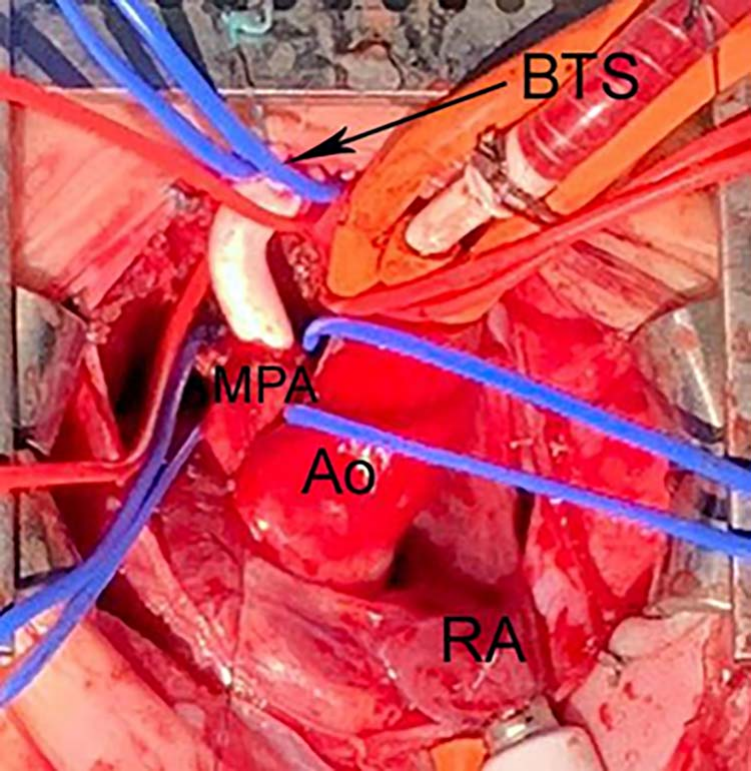
Intraoperative photograph shows Blalock-Taussig shunt between right BA and MPA. The PDA was divided under CPB to allow space for implantation of the shunt. Ao, Aorta; CPB, cardiopulmonary bypass; BTS, Blalock-Taussig shunt; CPB, cardiopulmonary bypass; LAA, left atrial appendage.

The subsequent pre-operative computed tomography angiography (CTA) thorax confirmed visceroatrial situs inversus, mirror image dextrocardia, tetralogy anatomy and left aortic arch with bovine branches. The MPA was hypoplastic (4 mm) with atretic origin. The right and left-sided pulmonary arteries measured 4 and 3 mm, respectively, as compared with the aorta (8 mm). A right PDA was seen originating from the brachiocephalic artery. The inferior part of the PDA was tortuous and connected to the proximal right pulmonary artery (Figs 2–4). At Day 20 of life, he underwent division of PDA, modified Blalock-Taussig shunt with Gore-Tex 3.5 mm between brachiocephalic artery (before bifurcation) and at the bifurcation of MPA through median sternotomy (Figs 5 and 6). The ascending aorta was twice the size of MPA, and the MPA was moderate in size (±3 mm). The right and left pulmonary arteries measured 4 and 3 mm, respectively. The tortuous and small PDA was noted to arise from the brachiocephalic artery, and it was connected to the proximal right pulmonary artery. Dissection of PDA has caused several episodes of hemodynamic instability, it was therefore decided to continue the procedure under normothermic cardiopulmonary bypass (CPB). It was easy weaning from cardiopulmonary bypass but with high doses of inotropes. In view of hemodynamic instability, the decision was made to leave the sternum open. He was transferred to the ICU in relatively labile condition, with high ventilator setting and inotropic support. He later developed pulmonary over circulation and remained intubated until Day 10 post surgery. He remained in the ICU and was discharged well on Day 26 post surgery. Serial post-operative echocardiography demonstrated good shunt flow with oxygen saturations maintained above 85% on air.

## DISCUSSION

Although TOF is a prevalent congenital anomaly, there are only few reports of its association with dextrocardia and situs inversus. The prevalence was 1.6% of the 3 anomalies together, which was first describe in 1952 [1, 2]. This case highlights a rare association TOF, dextrocardia and situs inversus, with concomitant unilateral right PDA and left aortic arch.

In the initial embryonic stage, both right and left sides of the arterial duct exist [3]. Usually, the left ductus can attach to either left or right pulmonary artery, or to any location on the aortic arch or brachiocephalic artery [4]. The right ductus constantly disappears [5]. In this case, we have observed the right-sided PDA was tortuous and S-shaped. This arch anomaly does not create a true vascular ring and not associated with symptomatic airway compression.

With this highly complex anatomy, we successfully performed the Modified Blalock-Taussig shunt under cardiopulmonary bypass.

The use of CPB for the Blalock-Taussig Shunt procedure is unavoidable because of the described anatomy and he was too unstable to tolerate the PDA dissection and continuation of procedure off-pump. Although the procedure generally predicts early death [6]; and the patient has relatively stormy post-operative event, he was discharged home well after 26 days in ICU.

## AUTHORS' CONTRIBUTIONS

KAS, AMS, AZM and MRMZ carried out the studies and drafted the manuscript and helped to draft the manuscript. NR, AMM and SA participated in the manuscript revision. All authors contributed to the article and approved the submitted version.
